# Supplementary material for: Effects of a dietary intervention on cardiometabolic risk and food consumption in a workplace
Source: PLoS One. 2024 Apr 24;19(4):e0301826. doi: 10.1371/journal.pone.0301826 (PMC11042715; doi:10.1371/journal.pone.0301826)
Supplement: S1 Table — (DOCX) [file pone.0301826.s001.docx]

Table 1: List of food added

| Food Groups | |
| --- | --- |
| Whole grains | **Rice Based:** Biryani (100%), Biryani (50%), Brown Rice (100%), Kheer (75%), Mixed Rice (50%), Pulao (50%) |
|  | **Corn Based:** Corn, Green Maize, Maize, Puri, Puri (75%), Popcorn |
|  | **Wheat Based:** Corn Flakes, Corn Powder, Wheat Flakes, Brown Bread |
|  | **Others:** Dhedo, Dosa |
| Refined grains | **Rice Based:** Biryani, Fried Rice, Jaulo, Kheer, Khichadi, Parboiled Rice, Pulao, Rice Pudding, White Rice, Sel |
|  | **Flour-Home:** Chamal Roti, Chatamari, Yomari, Nimki, Pani Puri, Pani Roti, Naan, Maida Roti, Pancake, White Bread, Sandwich |
|  | **Flour-Factory:** Bun, BreadChop, Doughnut, Banana Cake, Fruit Cake, Cake, Muffin, Puff, Toast, Cream Doughnut |
|  | **Beaten Rice:** Furandan, Murai, Chatpate, Beaten Rice |
|  | **Refined plus sweet:** Haluwa, Khajuri, Puwa, Sewai, Jery, Malpuwa |
|  | **Noodles:** Maggi, Mama Noodles, Ramen, Rara, Tenz Noodle, Thukpa, Waiwai, Patpate, Laphing, Macaroni, Pasta |
|  | **Momo:** Buff Momo, Chicken Momo, Kothey Momo, Veg Momo |
|  | **Chow Mein:** Buff Chowmein, Chicken Chowmein, Egg Chowmein, Egg Roll, Mixed Chowmein, Veg Chowmein |
|  | **Burger:** Chicken Burger, Veg Burger |
|  | **Others:** Samosa, Samosa Chaat, Kurkure, Dalmot |
| Potato | Aloo Achar, Aloo Paratha, Potato, Aloo Bhujia, Aloo Chop, Potato Chips, French Fries, Aalu Chips, PotatoChips, FrenchFries, AaluChips, Tarul, Pidalu |
| Vegetables | Bakula, Barela, BeetRoot, Brinjal, Broccoli, Capsicum, Carrot, CarrotAchar, Cabbage, Chauli, ChauliChana, Chichindo, Cucumber, CucumberAchar, Eskus, Lauka, Ghiraula, Karela, Lady Finger, Parwal, Karkalo, Green Bodi, Green Peas, Green Simi, Green Soybean, Koirala, Kurilo, Pumpkin Stem, Saag, Sisnu, Eskus Stem, Ground Apple, Gundruk, JackFruit, Junar, Mushroom, Mushroom Chilli, Mushroom Choila, Mushroom Soup, Nimbu Achar, MixedCurry, MulaAchar, Niguro, Onion, Peas, Raddish, Pakora, Pumpkin, Taruwa, Tomato, Tomato Achar, Tomato AcharC, Veg Burger, Veg Momo, Veg Soup, Veg Tampura, Momo Achar, Momo Pickle |
| Fruits | Apple, Avocado, Banana, Bhogate, Amala, Lemon, Lemon Achar, Orange, Grapes, Lapsi Achar, Lapsi Mada, Guava, Mango, Mausam, Naspati, Lichi, Papaya, Peach, Plum, Pomegranate, Pineapple, Raspberry, Sarifa, Watermelon, Pudina Achar |
| Fats Mono/Poly | Corn Oil, Mustard Oil, Olive Oil, Soybean Oil, Sunflower Oil |
| Fats saturated | Butter, Cream, Cream Doughnut, Cheese, Chhurpi, Garlic Cheese, Ghee |
| Lentils | Bara, Beans, Bhatmas, BhatmasDhulo, Daal Chana, Daal Gahat, Daal Maas, Daal Masuri, Daal Masyang, Daal Moong, DaalRahar, Daal Simi, Kalo Daal, Mixed Daal, Fried Papad, Fulaura, Masala Papad, Masaura, Mass Masyaura, Masyaura, Moong Roti, Papad Puri, Rajma, Roasted Bokula, Bodi, Qwati |
| Nuts | Almond, Kaju, Kismis, Chhokada, Dates, Coconut, Peanut Sadeko, Peanut, Peanut Butter, Roasted Peanut, Pistachio, Walnut, Pumpkin Seed |
| Red meat | Badel Meat, Battai Meat, Bhutan, Keema, Buff Brain, Buff Choila, Buff Chilli, Buff Fried, Buff Gravy, Buff Jibro, Buff Kachila, Buff Sausage, Buff Sukuti, Kachila, Buff Momo, Buff Chow mein, Mutton Fry, Mutton Gravy, Mutton Soup, Pork Meat |
| White meat | Chicken Burger, Chicken Chow mein, Chicken Momo, Chicken Soup, Egg Roll, Egg Chowmein, Kothey Momo, Boiled Egg, Egg Curry, Egg Whites, Omelette, Chhoela, Chicken Barbeque, Chicken Chilli, Chicken Drumsticks, Chicken Choila, Chicken Fry, Chicken Gravy, Chicken Meatball, Chicken Roast, Chicken Sausage, Dry Meat, Pangra, Rakti Blood, Tandoori |
| Fish | Fish Chilli, Fish Curry, Fish Finger, Fish Fry, Tuna |
| Sugar-sweetened Beverages (SSB) | Cold Drinks, Boost, Horlicks, Aloe Vera Juice, Amla Juice, Karela Juice, Orange Juice, Pineapple Juice, Real Juice,  Litchi Juice, Tang Juice, Tang Powder, Banana Lassi, Lassi, Oreo Smoothie, ORS |
| Other Beverages (Tea/Coffee) | Black Coffee, Cold Coffee, Milk Coffee, Barley Tea, Milk Tea, Black Tea, Tea Sarbottam Pitho, Green Tea, Hot Lemon, Lemon Tea, Lemon Tea Honey |
