# Supplementary material for: Effects of a dietary intervention on cardiometabolic risk and food consumption in a workplace
Source: PLoS One. 2024 Apr 24;19(4):e0301826. doi: 10.1371/journal.pone.0301826 (PMC11042715; doi:10.1371/journal.pone.0301826)
Supplement: S2 Table — (DOCX) [file pone.0301826.s002.docx]

S2 Table. Comparison of characteristics of participants who completed the study and who dropped out of the study.

| **Characteristics** | **Completed the study**  **(n = 257)**  **n (%)** | **Drop out from the study**  **(n = 56)**  **n (%)** | **P-value** |
| --- | --- | --- | --- |
| **Age, years Mean (SD)** | 31.1±7.6 | 37.9±8.6 | <0.0001 |
| **Gender** |  |  |  |
| Male | 105(40.9) | 16(28.6) | 0.087 |
| Female | 152(59.1) | 40(71.4) |  |
| **Ethnicity** |  |  |  |
| Brahmin/Chhetri | 93(36.2) | 21(37.5) | 0.539 |
| Newar | 122(47.5) | 30(53.6) |  |
| Tamang | 29(11.3) | 3(5.4) |  |
| Others | 13(5.1) | 2(3.6) |  |
| **Marital Status** |  |  |  |
| Married | 175(68.1) | 45(80.4) | **0.003** |
| Not Married | 82(31.9) | 11(19.6) |  |
| **Religion** |  |  |  |
| Hindu | 229(89.1) | 52(92.9) | 0.816 |
| Not Hindu | 28(10.9) | 4(7.1) |  |
| **Education** |  |  |  |
| Less than high school | 73(28.4) | 15(26.8) | 0.807 |
| High School or more | 184(71.6) | 42(73.2) |  |
| **Years of education, Mean (SD)** | 12.7±3.8 |  |  |
| **Smoking** |  |  |  |
| Never | 198(77.0) | 42(75.0) | 0.805 |
| Former | 17(6.6) | 3(5.4) |  |
| Current | 42(16.3) | 11(19.6) |  |
| **Alcohol intake (drinks per day)** |  |  |  |
| Never | 121(47.1) | 26(47.3) | 0.304 |
| 1 or less drinks per day | 112(43.5) | 24(43.6) |  |
| 1- 2 drinks per day | 11(4.3) | 0 |  |
| 3 or more drinks per day | 13(5.1) | 5(9.1) |  |
| **Physical activity** |  |  |  |
| Low (<600 METmin/ week) | 93(41.5) | 13(27.7) | 0.077 |
| High (600 or more METmin/week) | 131(58.5) | 34(72.3) |  |
| **Body Mass Index** |  |  |  |
| Underweight (18.5 kg/m2 or less) | 4(1.6) | 1(1.8) | **0.008** |
| Normal (18.6 – 24.9 kg/m2) | 139(54.9) | 17(30.4) |  |
| Overweight (25.0 – 29.9 kg/m2) | 94(36.6) | 34(60.7) |  |
| Obesity (30 kg/m2 or more) | 20(7.8) | 4(7.1) |  |
| **Blood pressure** |  |  |  |
| Normal (< 120/80 mmHg) | 76(29.6) | 56(100.0) | - |
| Prehypertension (120-139/ 80-89 mmHg) | 147(57.2) | 0 |  |
| Hypertension (>140/90 mmHg) | 34(13.2) | 0 |  |
| **Glycated hemoglobin (HbA1c)*** |  |  |  |
| Normal (<5.7%) | 188(80.3) | 23(41.9) | **<0.001** |
| Prediabetes (5.7- 6.5%) | 46(19.7) | 33(58.9) |  |
