# Supplementary material for: Effects of a dietary intervention on cardiometabolic risk and food consumption in a workplace
Source: PLoS One. 2024 Apr 24;19(4):e0301826. doi: 10.1371/journal.pone.0301826 (PMC11042715; doi:10.1371/journal.pone.0301826)
Supplement: S2 File — (DOCX) [file pone.0301826.s005.docx]

Harvard T.H. Chan School of Public Health

Office of Human Research Administration

90 Smith Street, 3rd Floor Boston, MA 02120

# Notification of Initial Study Approval

| April 25, 2016  Donna Spiegelman stdls@hsph.harvard.edu |  |
| --- | --- |
| **Protocol Title:** | Nepal Pioneer Worksite Intervention Study |
| **Principal Investigator:** | Donna Spiegelman |
| **Protocol #:** | IRB16-0301 |
| **Funding Source:** | None |
| **Review Date:** | 4/25/2016 |
| **STUDY Effective Date:** | 4/25/2016 |
| **Expiration Date:** | 4/24/2017 |
| **IRB Review Type:** | Expedited |
| **IRB Review Action:** | Approved |

On 4/25/2016, the Institutional Review Board (IRB) of the Harvard T.H. Chan School of Public Health approved this Initial Study. **Please note that the approval for this protocol will lapse on 4/24/2017.**

This approval includes the following:

- Initial Application, IRB16-0301
- IRB Protocol: nepal pioneer PROTOCOL DH_version2 _clean.doc (0.01)
- Consent Form: Nepal pioneer consent form CLEAN version 4-22.pdf (0.01)
- Recruitment Materials: Nepal pioneer invitation letter DH-version2_clean.pdf (0.01)
- Study Instrument/Tools: Identifiers.pdf (0.01)
- Study Instrument/Tools: baseline questionnaire_section2.pdf (0.01)
- Study Instrument/Tools: IDRS - English.pdf (0.01)
- Study Instrument/Tools: Baseline questionnaire - 1.pdf (0.01)
- Study Instrument/Tools: Baseline questionnaire_section1.pdf (0.01)

Additionally, the IRB has reviewed the following documents:

- Ancillary Approvals/Permissions: nepal pioneer - letter of support - DH.pdf (0.01)
- Other: whole-grain-painting.pdf (0.01)
- Other: WHD_2013_WHD_2013_Poster5.pdf (0.01)
- Other: whole grains_poster.pdf (0.01)
- PI's Current CV (ICH-GCP E6 Only): CV_Donna Spiegelman.pdf (0.01)
- Translation Attestation Form: nepal pioneer - translation attestation form - DH - signed.pdf (0.01)

University Area IRB [http://cuhs.harvard.edu](http://cuhs.harvard.edu/)

Longwood Medical Area IRB <http://www.hsph.harvard.edu/ohra/>

The IRB made the following determinations:

- Waivers: Waiver/alteration of the consent process
- Risk Determination: No greater than minimal risk
- Harvard Special Determinations: None
- Research Information Security Level: The research is classified, using Harvard’s Data Security Policy, as Level 3 Data.

The IRB requests the following:

- The Department Chair Review referenced funding from a Pioneer Grant. Please submit a modification to add the grant to Funding Source page.
- Upload approval from the ERB of Nepal Health Research Council into ESTR when it becomes available. Note that no human research activities can occur in the field until local review has been secured.
- Copies of locally-approved translated versions or a status update must be submitted to the IRB within 45 days of this notification letter.
- As a result of the IRB’s Data Security determination that the research is classified as Level 3 Data, please complete and submit a Level 3 worksheet (available at <http://vpr.harvard.edu/pages/harvard-research-data-security-policy>) to your local IT information security officer (HSPH aross@hsph.harvard.edu ). Please submit this signed worksheet (or documentation of IT approval) to the IRB before the study’s next annual renewal period.

Please contact me at 617-432-2160 or kturner@hsph.harvard.edu with any questions.

Sincerely,

Keisha Turner

IRB Review Specialist

University Area IRB [http://cuhs.harvard.edu](http://cuhs.harvard.edu/)

Longwood Medical Area IRB <http://www.hsph.harvard.edu/ohra/>
