# Supplementary material for: Effects of a dietary intervention on cardiometabolic risk and food consumption in a workplace
Source: PLoS One. 2024 Apr 24;19(4):e0301826. doi: 10.1371/journal.pone.0301826 (PMC11042715; doi:10.1371/journal.pone.0301826)
Supplement: S3 File — (DOCX) [file pone.0301826.s006.docx]

## Background

The burden of non-communicable diseases (NCDs), such as cardiovascular disease (CVD), diabetes, cancer, and chronic obstructive pulmonary disease (COPD), are on the rise in low- and middle- income countries [[1](https://bmccardiovascdisord.biomedcentral.com/articles/10.1186/s12872-019-1025-3#ref-CR1)]. CVD is the leading cause of morbidity, mortality, and disability in South Asia, where 20% of the world’s population resides [[2](https://bmccardiovascdisord.biomedcentral.com/articles/10.1186/s12872-019-1025-3#ref-CR2)].

Sedentary lifestyle, poor diet, and excessive body weight are reported to have a large effect on the risk of developing NCD’s [[3](https://bmccardiovascdisord.biomedcentral.com/articles/10.1186/s12872-019-1025-3#ref-CR3), [4](https://bmccardiovascdisord.biomedcentral.com/articles/10.1186/s12872-019-1025-3#ref-CR4)]. Lifestyle interventions addressing diet and exercise have reduced cardiovascular risk [[5](https://bmccardiovascdisord.biomedcentral.com/articles/10.1186/s12872-019-1025-3#ref-CR5), [6](https://bmccardiovascdisord.biomedcentral.com/articles/10.1186/s12872-019-1025-3#ref-CR6)]. Despite the evidence supporting the use of lifestyle interventions to prevent hypertension and diabetes and to improve glucose tolerance, their translation into real world settings has been challenging. Worksites provide unique opportunities for health promotion and disease prevention programs since many people spend a majority of their time at work, and they allow access to large segments of the population. Worksites also provide an infrastructure and natural environment for social support. Worksite-based health programs have shown positive impacts on employee health [[7](https://bmccardiovascdisord.biomedcentral.com/articles/10.1186/s12872-019-1025-3#ref-CR7), [8](https://bmccardiovascdisord.biomedcentral.com/articles/10.1186/s12872-019-1025-3#ref-CR8)], and they have led to significant improvements in cardiovascular risk factor profiles [[9](https://bmccardiovascdisord.biomedcentral.com/articles/10.1186/s12872-019-1025-3#ref-CR9), [10](https://bmccardiovascdisord.biomedcentral.com/articles/10.1186/s12872-019-1025-3#ref-CR10)]. Worksite interventions encompassing environmental changes (i.e. low-cost healthy food options), places for physical activity (i.e. fitness centers or gyms), and group-based health education classes, have been highlighted as components of successful worksite interventions [[11](https://bmccardiovascdisord.biomedcentral.com/articles/10.1186/s12872-019-1025-3#ref-CR11), [12](https://bmccardiovascdisord.biomedcentral.com/articles/10.1186/s12872-019-1025-3#ref-CR12)]. However, much of these evidences come from high income countries and there is limited studies from low income setting.

Thus, we planned a study that will measure the effectiveness of a cafeteria-based intervention (adding healthy foods and reducing or removing unhealthy foods), combined with a behavioral intervention, on cardio-metabolic risk among employees of a hospital based in Nepal, by evaluating the change in number of individuals reaching two or more cardio-metabolic risk goals, specifically reductions in blood pressure, triglycerides, and glycosylated hemoglobin (HbA1c).

If proven effective, the cafeteria, behavioral or both interventions can be scaled up in the worksites in Nepal. According to the Labor Force Survey 2008, the labor force comprises of 12 million people with a participation rate of 83.4%. An estimated 3 million adults work in formal employment including legislators, professionals, technicians, clerks, service workers, market agriculture, craft and trade, machine operators and armed forces [[13](https://bmccardiovascdisord.biomedcentral.com/articles/10.1186/s12872-019-1025-3#ref-CR13)]. Hence, the worksite based health promotion programs have potential to impact on the population health at large in Nepal.

## Methods

### Study design

The Nepal Pioneer Worksite Intervention Study is a two stage intervention study. In the first stage, we will use a pre-post design to assess how a cafeteria intervention that provides a healthier diet, affects cardio-metabolic risk. In the second step, we will conduct an unmasked two-arm randomized trial by allocating half of the participants to continue the cafeteria intervention in addition to a behavioral (CB) intervention for the prevention of cardio-metabolic risk, while the other half of participants will receive the cafeteria-only (CO) intervention. (Fig. [1](https://bmccardiovascdisord.biomedcentral.com/articles/10.1186/s12872-019-1025-3#Fig1)) The study protocol has been approved by the institutional review committee at Harvard T.H. Chan School of Public Health, Nepal Health Research Council, and Kathmandu University School of Medical Sciences. The study has been retrospectively registered in [clinicaltrials.gov](http://clinicaltrials.gov/) (Identification Member: NCT03447340) on February 27, 2018.

Fig. 1


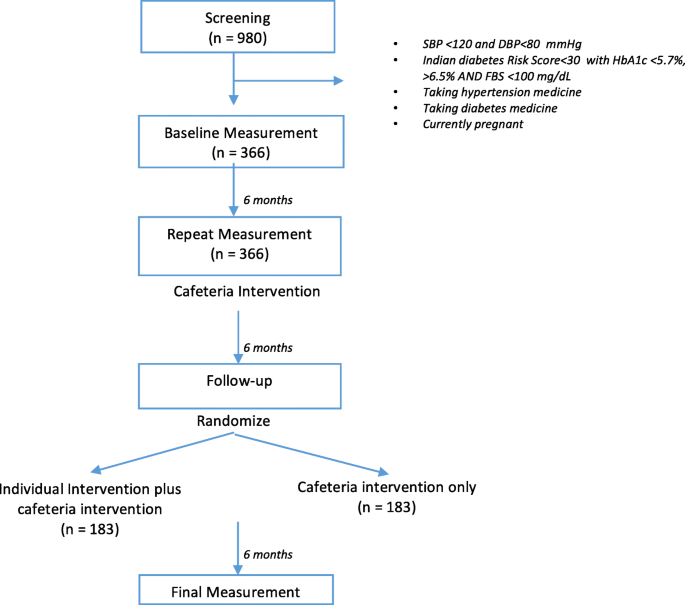


Planned flow of participants through the Nepal Pioneer Worksite Intervention Trial

### Study setting

The study will be conducted at Dhulikhel Hospital - Kathmandu University Hospital (DH-KUH) in central Nepal. Dhulikhel Hospital is an independently owned, not-for-profit institution which was conceived and supported by the Dhulikhel community. The hospital has approximately1040 employees and four cafeterias that are in operation 16 hours a day. There are no gyms or other facilities to help physical activity onsite. The hospital does not currently offer any wellness programs for the prevention of cardiometabolic diseases.

### Recruitment

We will conduct a 20-min information session in all of the hospital departments. Employees will be invited to attend sessions by flyers posted around the hospital and through announcements in department meetings. During the information session, we will explain the purpose and the expectations of the study. We will explain the ethical considerations, emphasizing the importance of protecting participant’s privacy during research-related interactions and outcomes, the volunteer nature of the study, the option to drop out of the study at any time, and we will establish that there will not be any coercion from supervisors to participate in the study. After the information session, research assistants (RAs) will set up appointments for eligibility screening among interested participants.

The inclusion criteria are: (a) adults 18 years or older; (b) full time employees of DH-KUH; with (c) systolic blood pressure of ≥120 mmHg or diastolic pressure ≥ 80 mmHg; or HbA1c of 5.7 to 6.4%, or fasting blood sugar of ≥100 mg/dL. The exclusion criteria of the study are: (a) pregnant women, since dietary habits may change during pregnancy, (b) taking diabetes medication, or (c) taking hypertension medication. Those employees who are taking medication receive free drugs and lifestyle counselling in the hospital’s diabetes and hypertension clinic. The RA will obtain written informed consent in a private room for those individuals who are deemed eligible to participate in the study. A short oral consent process will be utilized for participants who cannot read the consent form. This will entail presenting all of the elements of the consent form verbally to the participant, in the presence of a witness. The witness will be required to sign a document stating the consent form has been verbally presented to the participant.

### Data collection

The schedule of enrollment and assessment is presented in Table [1](https://bmccardiovascdisord.biomedcentral.com/articles/10.1186/s12872-019-1025-3#Tab1). At baseline, we will measure demographics, anthropometry, blood pressure, lifestyle, blood sugar and lipid profile. After 6 months of control period (without any intervention), we will measure the anthropometry, lifestyle, blood sugar and lipid profile. We will implement the cafeteria intervention for 6 months and re-measure the anthropometry, lifestyle, blood sugar and lipid profile at 12 months. Then, we will randomize half of the participants to continue the cafeteria intervention in addition to a behavioral intervention for the prevention of cardio-metabolic risk, while the other half of participants will receive the cafeteria-only intervention. We measure the anthropometry, lifestyle, blood sugar and lipid profile at 18 months.

Table 1 The schedule of enrollment and assessments for the Nepal Pioneer Worksite Intervention Trial

|  | Screening Month 1 | Baseline  Month 1–2 | Follow up - 1  Month 6 | Follow up- 2  Month 12 | Follow up- 3  Month 18 |
| --- | --- | --- | --- | --- | --- |
| Indian Diabetes risk score questions (age, sex, family history of diabetes self-report moderate physical activity) | X |  |  |  |  |
| Demographic information (age, sex, ethnicity, education, income, religion, marital status) |  | X |  |  |  |
| Anthropometry | | | | | |
| Height |  | X |  |  |  |
| Weight |  | X | X | X | X |
| Waist circumference | X | X | X | X | X |
| Hip circumference |  | X | X | X | X |
| Blood Pressure | X | X | X | X | X |
| Lifestyle factors | | | | | |
| Two 24 Hour Dietary recall |  | X | X | X | X |
| Global physical activity questionnaire |  | X | X | X | X |
| Smoking and Drinking habits |  | X |  | X |  |
| Laboratory | | | | | |
| Glycated hemoglobin | X | X | X | X | X |
| Fasting Blood Sugar | X | X | X | X | X |
| High density lipoprotein cholesterol |  | X | X | X | X |
| Low density lipoprotein cholesterol |  | X | X | X | X |
| Triglycerides |  | X | X | X | X |
| Total Cholesterol |  | X | X | X | X |

#### Screening

The RAs will conduct a 2 phased screening process to identify eligible participants. In the first screening, the RAs will screen participants by measuring blood pressure and administering a questionnaire along with measures that it make it possible to calculate the Indian Diabetes Risk Score (IDRS) [[14](https://bmccardiovascdisord.biomedcentral.com/articles/10.1186/s12872-019-1025-3#ref-CR14)]. The IDRS takes age, abdominal obesity, self-reported physical activity, and family history of diabetes into account. The IDRS score ranges from 0 to 100, with a high number indicating higher risk. The IRDS has been considered a reliable instrument to screen the risk of diabetes in Asian-Indian populations [[14](https://bmccardiovascdisord.biomedcentral.com/articles/10.1186/s12872-019-1025-3#ref-CR14)]. An IDRS of 30 or more has been shown to have 95% sensitivity and 45% specificity to detect prediabetes using fasting blood sugar criteria (100–120 mg/dL); and 87% sensitivity and 47% specificity using HbA1c criteria (HbA1c 5.7–6.5%) in an analysis of 560 free residents of Dhulikhel (Unpublished).

Blood pressure will be measured in the right arm of seated participants, after a five- minute rest period. Three measurements of systolic and diastolic blood pressure will be taken using a Microlife automatic blood pressure measuring device. The mean of three blood pressure measurements will be used. Participants with systolic blood pressure of ≥120 mmHg or diastolic pressure ≥ 80 mmHg will be invited to participate regardless of their IDRS score. In the second screening, the participants scoring 30 or more on IDRS will be asked to provide a blood sample to measure HbA1c and fasting blood glucose. The individuals with HbA1c between 5.7 to 6.4% or fasting blood sugar of ≥100 mg/dL will be invited to participate. All eligible individuals that provided informed consent will be enrolled. No compensation or reimbursement will be provided to the participants.

#### Baseline assessments

At baseline, RAs will interview the participants using a standardized electronic questionnaire using Open Data Kit software [[15](https://bmccardiovascdisord.biomedcentral.com/articles/10.1186/s12872-019-1025-3#ref-CR15)]. RAs will receive 2 weeks training on data collection and ethical issues.

The questionnaire will assess socioeconomic characteristics including age, sex, ethnicity, religion, marital status, annual income, education, and lifestyle factors including smoking, alcohol intake, and physical activity. We will use the Global Physical Activity Questionnaire [[16](https://bmccardiovascdisord.biomedcentral.com/articles/10.1186/s12872-019-1025-3#ref-CR16)], and calculate the metabolic equivalent of task (MET) minutes per week. A weekly MET equivalent of 600 would represent 30 min of brisk walking five times per week or 15 min of running five times per week.

#### Twenty four hour diet recall

To measure dietary intake, we will conduct two interviewer-administered 24-h dietary recalls within a week. Each 24-h dietary recall will take approximately 25 min to complete. First, activities of the previous day will be documented to refresh the participant’s memory. Then, a dietitian will ask the participant to recall everything s/he consumed from the first meal to last meal. The time and place of each meal will be noted, followed by detailed information on each food including: specific brands, ingredients, and/or recipes. Participants will be asked to report their food portions using colorful examples of sizes or household measures such as spoon, bowl, etc. If a participant reports using their own recipe, then complete information on each individual ingredient will be inquired about. Energy and nutrient intakes will be calculated using the food composition table for Nepal [[17](https://bmccardiovascdisord.biomedcentral.com/articles/10.1186/s12872-019-1025-3#ref-CR17)].

#### Anthropometry

Body weight will be measured with minimum clothing and without shoes using an Omron Model HBF-400 scale and recorded to the nearest 0.1 pounds. The weighing scale will be calibrated to zero every day. Participants’ heights will be measured, without shoes, while the participants stand against a wall. Height will be measured using a tape measure and recorded to the nearest 0.1 cm.

#### Laboratory

Blood samples will be analyzed for HbA1c, fasting glucose, low density lipoprotein (LDL) cholesterol, high density lipoprotein (LDL) cholesterol, triglycerides, and total cholesterol. All of the laboratory procedures will be carried out in the biochemistry laboratory of DH-KUH. Blood samples will be collected using evacuated blood collection tubes. Participants will be asked to fast overnight (8–14 h). We will record the time of blood draw and their most recent shift worked prior to the blood draw. The HbA1c will be measured using Boronate affinity chromatography (Axis-Shield, Norway) [[18](https://bmccardiovascdisord.biomedcentral.com/articles/10.1186/s12872-019-1025-3#ref-CR18)]; fasting blood glucose using Hexodinase method (Dialab, Austria) [[19](https://bmccardiovascdisord.biomedcentral.com/articles/10.1186/s12872-019-1025-3#ref-CR19)]; LDL and HDL using the elimination method (Dialab, Austria) [[20](https://bmccardiovascdisord.biomedcentral.com/articles/10.1186/s12872-019-1025-3#ref-CR20)]; triglyceride using GPO-PAP (Dialab, Austria) [[21](https://bmccardiovascdisord.biomedcentral.com/articles/10.1186/s12872-019-1025-3#ref-CR21)]; and total cholesterol using CHOD-PAP (Dialab, Austria) [[22](https://bmccardiovascdisord.biomedcentral.com/articles/10.1186/s12872-019-1025-3#ref-CR22)]. For each type of assay, the laboratory has quality control (QC) materials (using commercially available assayed and unassayed control material) from Bio-Rad Laboratories, USA. Each QC is run at least in duplicate. External QC is arranged by internationally recognized reference laboratories that distribute batches of samples of various concentrations for each assay. The laboratory performs the External Quality Assurance Scheme from unknown assayed sample from the Department of Clinical Biochemistry CMC, Vellore, India for 23 routine parameters, 5 immunological parameters and HbA1c. Additionally, 5% of the blood samples will be obtained in duplicates and sent for testing all parameters, blinded to the laboratory personnel.

#### Control period

We will have a control period of 6 months before implementing the intervention. There will not be any contact with the enrolled participants until 6 months after the study enrollment. The control period will minimize potential biases in effect estimates, including increases or decreases in a health condition with time.

### Interventions

#### Step 1: Cafeteria intervention

After 6 months of the control period, participants will receive the cafeteria intervention (Table [2](https://bmccardiovascdisord.biomedcentral.com/articles/10.1186/s12872-019-1025-3#Tab2)). The cafeteria intervention was developed based on the findings from four focus group discussions with cafeteria users and nine in-depth interviews with cafeteria operators and managers, about strategies to promote healthy foods in the worksite. (unpublished) The four cafeterias in the hospital will improve the quality of their meals by (1) increasing the availability of fresh fruit (not fruit juice) and vegetable options, (2) avoiding sales of sugar sweetened beverages, (3) replacing whole grains with refined grains in cooking; (4) using healthy vegetable oils such as soy and sunflower; (5) minimizing the sale of fried foods; (6) trimming animal fats from meats before cooking; (7) using healthier protein sources such as chicken, beans, and nuts (8) making potable water free of cost; and (9) reducing salt in cooking. These guidelines are based on the recommendations for a healthy diet to improve cardiovascular health [[23](https://bmccardiovascdisord.biomedcentral.com/articles/10.1186/s12872-019-1025-3#ref-CR23)]. To facilitate these changes, a cafeteria operation team will be formed and they will be trained on procedures to implement, supervise, and monitor the worksite’s healthy changes. In addition, we will train the cafeteria staff on healthy eating, and how to modify recipes to incorporate healthy options.

Table 2 Cafeteria Intervention in the Nepal pioneer worksite intervention study

| Components | Activities |
| --- | --- |
| Strengthen management of cafeteria | • A cafeteria improvement team will be created representing hospital management, cafeteria operation, research team, finance department, hygiene monitoring, non-communicable disease prevention, nutritionist, and human resource department  • We will conduct a 2-h workshop with the team on the prevention of diabetes, hypertension, and other cardiovascular diseases via healthy eating.  • The team will meet monthly to monitor the intervention; discuss and find solutions for barriers to implementation |
| Build capacity of cafeteria staff | • We will conduct a 2-h workshop with the cafeteria staff on the prevention of diabetes, hypertension, and other cardiovascular diseases via healthy eating  • We will conduct two sessions of two-hour workshops on healthy cooking practices with the cafeteria staff |
| Replace healthy options for unhealthy foods | • Whole grain bread instead of white bread will be used in cooking bread omelets, or fried bread  • Peanut butter will be replaced for jam  • White rice will be mixed with 50% brown rice in plain rice, fried rice, and rice pudding recipes  • Whole wheat flour will be mixed with all-purpose flour in 1:4 ratio in dumpling recipes  • Additional vegetables and lentils will be added in chowmein, thukpa, fried rice  • Lentils (black beans) will be used with beaten rice instead of potato |
| Remove unhealthy foods from the cafeteria | • Soda drinks (Coke, Fanta, Sprite, Pepsi, Mountain Dew) will be removed  • High sugar confectioneries (cake, donut, puff, cream-donut, biscuits) will be removed |
| Add healthy food options | • Fresh fruits in the form of whole fruit and mixed fruit salad  • Flavored water free of cost  • Salad (cucumber, radish, carrots)  • Whole grains (oats, whole wheat bread, popcorn) |
| Enhance visibility of healthy foods | • Healthy food options will appear in the front page of the electronic menu  • Fruits, salads and whole grains will be organized on the front desk counter |
| Information, Education, Communication | • A formal launch program will be organized; all the department heads of the hospital will be invited to attend  • A kickoff event will be organized in each cafeteria, with a kiosk providing information on eating healthy in the cafeteria  • Posters showing the healthy eating plate and whole grain consumption will be displayed on the cafeteria walls  • All employees in all of the departments of the hospital will be invited to attend a 20 min session on making healthy choices in the cafeteria |

#### Step 2: The behavioral intervention

After 6 months of the cafeteria intervention, half of the participants will be randomized using computer generated random numbers to receive a cafeteria and behavioral intervention (CB), the other half of the participants will continue to receive the cafeteria only (CO) intervention. The behavioral intervention will be comprised of intensive education sessions, group counseling, and goal setting and monitoring exercises based on the Diabetes Prevention Program (DPP) [[24](https://bmccardiovascdisord.biomedcentral.com/articles/10.1186/s12872-019-1025-3#ref-CR24)] tailored to local needs. The curriculum includes 24 sessions of 16 core weekly sessions during the first 4 months of this phase of the intervention followed by 8 weekly maintenance sessions. Each session will be 1 h long, and will be facilitated by a dietitian. Broadly, the curriculum covers subject matters of importance for achieving and maintaining healthy weight, eating a healthy diet, increasing physical activity, stress management, and overcoming common challenges encountered when making lifestyle changes. Similar to the cafeteria intervention, healthy eating messages include increasing fresh fruits and vegetable intake, avoiding added sugar, choosing whole grains, choosing healthier sources of protein, reducing sodium, and monitoring portion sizes. Participants will be encouraged to keep food and activity diaries throughout the course of the study. The maintenance period’s focus will be on overcoming declines in motivation and maintaining long-term healthy behaviors. All of the sessions will be conducted at the worksite during the workday. There will be about 20 participants in each education class. Participants will set a minimum of two lifestyle-change goals (e.g. Half of their total grain intake will be whole grains, walking 30 min a day, or reducing 7% of their body weight).

### Follow up

We will assess outcomes at 6 months (at the end of the control period), 12 months (at the end of the cafeteria intervention), and 18 months (at the end of the behavioral intervention). During each follow up, fasting blood samples will be collected and analyzed for HbA1c, fasting glucose, and lipid profile (HDL, LDL, total cholesterol, triglycerides). We will re-administer the global physical activity questionnaire, and the two 24-h diet recalls. We will re-measure height, weight, waist circumference, and blood pressure at each of these time points as well.

### Primary and secondary outcomes

The primary outcome will be the proportion of individuals reaching two or more of their cardio-metabolic risk goals, reductions in blood pressure, triglycerides, and HbA1c. Participants will be scored on the number of improved risk factors (0–3) as defined by decreases in (1) HbA1c ≥0.5%; (2) systolic blood pressure ≥ 5 mmHg; or (3) plasma triglycerides ≥10 mg/dl. These outcomes were selected because blood pressure, HbA1c, and triglycerides are commonly measured in clinical settings. This makes their use clinically appropriate and translatable, especially since other CVD risk scores such as the Framingham Risk Score, do not perform well in South Asian populations [[25](https://bmccardiovascdisord.biomedcentral.com/articles/10.1186/s12872-019-1025-3#ref-CR25)]. Moreover, the composite outcome allows for individuals to reduce different factors based on their variable risk profiles at baseline. For example, an individual with a baseline systolic blood pressure of 120 mmHg many not reduce this risk factor by as much as 5 mmHg, but may succeed in reducing HbA1c or triglycerides. The secondary outcomes are absolute changes in HbA1c, systolic blood pressure, diastolic blood pressure, and triglycerides individually.

### Data management

We have taken four robust provisions to ensure data quality. First, the electronic questionnaire will be closed only if the data collection is complete to avoid partial or missing values. The data will be cleaned and checked every month. The answer fields for all integer variables will be constrained to ensure entry of only valid numbers. Second, RAs will receive intensive training on data collection and ethical considerations. Third, the site investigator will supervise the RAs on a day-to-day basis. Fourth, the principal investigators will hold weekly meetings with site investigators, and if required, with the RAs, to discuss the course of the intervention and to address any issues.

#### Data analysis plan

The primary analysis will be intention to treat. The quantitative data analysis will follow the Consolidated Standards of Reporting Trials (CONSORT) guidelines [[26](https://bmccardiovascdisord.biomedcentral.com/articles/10.1186/s12872-019-1025-3#ref-CR26)]. The flowchart will include the number of participants seen at each stage, including the number screened, eligible, randomized, and analyzed for the primary outcomes.

#### Effectiveness of the cafeteria intervention

At the 12-month follow-up, we will compare the proportion of participants who achieved two or more of their cardio-metabolic risk factor reduction goals during the cafeteria intervention period, to the proportion of participants in the control period, using the generalized estimating equations (GEE) approach with the binary distribution, logit link function and an exchangeable working correlation structure [[27](https://bmccardiovascdisord.biomedcentral.com/articles/10.1186/s12872-019-1025-3#ref-CR27)].

#### Effectiveness of the behavioral intervention

At the 18-month follow-up, we will compare the proportion of participants who have achieved two or more of their cardio-metabolic risk factor reduction goals in CO arm to the proportion of participants in the CB arm using a *χ*2 test. In the event that randomization does not appear to have controlled for differences between the treatment and control groups on baseline characteristics, in secondary analysis, we will statistically control for those differences using the GEE approach with the binary distribution, logit link function and an exchangeable working correlation structure [[27](https://bmccardiovascdisord.biomedcentral.com/articles/10.1186/s12872-019-1025-3#ref-CR27)].

Our secondary analysis will compare the change in HbA1c, systolic blood pressure, and lipids during the cafeteria-only intervention to the change during the control period using a paired t-test. Similarly, we will compare the change in HbA1c, systolic blood pressure, and lipids between CO group and CB group using a paired t-test. Assumptions of the statistical tests will be examined and if violated non-parametric testing will be explored. In the event that randomization does not appear to have controlled for differences between the treatment and control groups in baseline characteristics, we will conduct the GEE approach with the Gaussian distribution, identity link function and an exchangeable working correlation matrix [[27](https://bmccardiovascdisord.biomedcentral.com/articles/10.1186/s12872-019-1025-3#ref-CR27)] to adjust for potential confounding factors. The data will be analyzed using STATA-15.

### Sample size and power

The pre-post design, with at least 366 eligible and enrolled participants [[28](https://bmccardiovascdisord.biomedcentral.com/articles/10.1186/s12872-019-1025-3#ref-CR28)] and 5% loss to follow-up (LTF) after 12 months, will have over 90% of power to detect the primary effectiveness endpoint of this trial, of 31.5% or greater, compared to the 21% change in the control group seem previously in a South Asian population [[29](https://bmccardiovascdisord.biomedcentral.com/articles/10.1186/s12872-019-1025-3#ref-CR29)]. For the analysis of two randomized groups (CO and CB arms), with 320 participants, we will be able to detect a relative risk of 1.5 given 0.315 risk probability in the control group, 5% LTF rate with 80% power and 5% level of significance [[30](https://bmccardiovascdisord.biomedcentral.com/articles/10.1186/s12872-019-1025-3#ref-CR30)].

In the secondary analysis, where we will compare the within-participant changes in HbA1c, systolic and diastolic blood pressure, and lipids observed in the cafeteria intervention to those observed in the behavioral intervention. Given the sample sizes of 366 and 320 respectively, 5% LTF rate with 80% power and 5% level of significance, we will be able to detect the minimum differences as presented in Table [3](https://bmccardiovascdisord.biomedcentral.com/articles/10.1186/s12872-019-1025-3#Tab3). For the before-after design, we considered three possible values of the correlation (*ρ* = 0.5, 0.6, 0.7) between repeated measurements during the control period and during the intervention period.

Table 3 Minimum absolute detectable differences for the change in glycosylated hemoglobin, systolic and diastolic blood pressure, and triglyceride for sample sizes of 366 and 320 correspondingly, 5% loss-to-follow-up rate with 80% power and 5% level of significance, for the Nepal Pioneer Worksite Intervention Trial

|  | | Minimum absolute detectable difference | | | |
| --- | --- | --- | --- | --- | --- |
|  |  | *N* = 366 | | | *N* = 320 |
| Parameter | Std. Dev. | *ρ*∗=0.5 | *ρ*∗=0.6 | *ρ*∗=0.7 | Independent samples |
| Glycated hemoglobin, % | 0.38 | 0.06 | 0.05 | 0.045 | 0.12 |
| Diastolic blood pressure, mmHg | 9.43 | 1.4 | 1.3 | 1.1 | 3 |
| Systolic blood pressure, mmHg | 13.32 | 2 | 1.8 | 1.6 | 4.3 |
| Triglycerides, mg/dL | 82.47 | 12.5 | 11.2 | 9.7 | 26.6 |

- *Correlation between the changes of the same individual observed during control and treatment periods of time
- Standard deviations are calculated from the baseline data of the study participants

### Minimization of contamination

The risk and level of contamination during the randomized study phase for the behavioral intervention will be monitored at the participant level. The participants will be instructed not to share information about the study and not to provide any support to people in their worksite, other than their classmates. The behavioral classes will be conducted in a separate building outside of their workspace to ensure privacy. Further, we will measure possible contamination by asking if the participants have received any information / advice regarding diet and lifestyle changes from any of their peers and if they did, collect the name and contact information of the peer. If the contamination is found to be significant, we will make adjustments by adding the contamination as a variable in the final model while estimating the effect in secondary analysis.

### Intervention fidelity

Intervention fidelity refers to the extent the intervention is delivered as it was intended [[31](https://bmccardiovascdisord.biomedcentral.com/articles/10.1186/s12872-019-1025-3#ref-CR31)]. In the proposed study, we will quantitatively assess the fidelity of both the cafeteria and the behavioral interventions. First, we will provide training sessions on the study objectives, details about the interventions, and the importance of fidelity to the study staff, cafeteria improvement committee and cafeteria staff. Second, we will monitor the implementation of changes in the cafeteria using a structured checklist on a weekly basis. The checklist is provided in the Additional file [1](https://bmccardiovascdisord.biomedcentral.com/articles/10.1186/s12872-019-1025-3#MOESM1). Any deviances observed in fidelity will be discussed at the monthly meetings of the cafeteria improvement team, where necessary actions will be planned and assessed. For the behavioral intervention, we will measure participant attendance, and we will test pre- and post- knowledge on diabetes prevention, as covered in the health education sessions. Finally, a site investigator will supervise the implementation of intervention.

### Interim analyses

We do not have a plan for interim analysis and do not expect a situation that would lead us to stop the study. Participants with newly reported pregnancy during the course of the study will be excluded from the study.

### Reportable events and indemnities

Serious adverse events are not expected. Other events related to study participation (such as an infection at the site of a blood draw clinic, or a breach of confidentiality) will be documented and appropriate remedial actions will be undertaken in a timely manner. All unanticipated non-medical problems that involve risk to subjects or others will be reported to the institutional review board at Kathmandu School of Medical Sciences and Harvard T.H. Chan School of Public Health.

## Discussion

We propose to implement and test a worksite-based lifestyle intervention program focusing on dietary improvement at the environmental and individual level in a hospital in Nepal. For primary prevention of CVD, an initial period of lifestyle modification is advocated, including improved diet and physical activity [[32](https://bmccardiovascdisord.biomedcentral.com/articles/10.1186/s12872-019-1025-3#ref-CR32)]. However, lifestyle change is not always easy. Both adoption and maintenance of new behaviors pose challenges for many individuals [[33](https://bmccardiovascdisord.biomedcentral.com/articles/10.1186/s12872-019-1025-3#ref-CR33), [34](https://bmccardiovascdisord.biomedcentral.com/articles/10.1186/s12872-019-1025-3#ref-CR34)].

Implementing health programs at worksites allows for continuous engagement with adults for positive and sustainable changes in lifestyle choices. Current evidence indicates that health and wellness programs at worksites provide numerous benefits with respect to altering cardiovascular risk factor profiles [[9](https://bmccardiovascdisord.biomedcentral.com/articles/10.1186/s12872-019-1025-3#ref-CR9), [10](https://bmccardiovascdisord.biomedcentral.com/articles/10.1186/s12872-019-1025-3#ref-CR10)]. Studies aimed at high-risk populations may have a larger health impact, and they may be more cost-effective than those targeting non-risk populations, because there is more room for improvement among high-risk populations [[35](https://bmccardiovascdisord.biomedcentral.com/articles/10.1186/s12872-019-1025-3#ref-CR35)].

Health promotion strategies that extend beyond education or communication can achieve significant behavioral changes [[9](https://bmccardiovascdisord.biomedcentral.com/articles/10.1186/s12872-019-1025-3#ref-CR9), [10](https://bmccardiovascdisord.biomedcentral.com/articles/10.1186/s12872-019-1025-3#ref-CR10)]. Environmental strategies aim to reduce barriers or increase opportunities for healthy choices, such as providing more healthy options, making healthy choices more accessible, and establishing policies that require healthy choices or restrict less healthy options [[36](https://bmccardiovascdisord.biomedcentral.com/articles/10.1186/s12872-019-1025-3#ref-CR36)]. Several literature reviews have evaluated the effectiveness of worksite health promotion trials. Most of these trials are individual-level interventions, and only a few contain environmental modifications [[7](https://bmccardiovascdisord.biomedcentral.com/articles/10.1186/s12872-019-1025-3#ref-CR7), [37](https://bmccardiovascdisord.biomedcentral.com/articles/10.1186/s12872-019-1025-3#ref-CR37), [38](https://bmccardiovascdisord.biomedcentral.com/articles/10.1186/s12872-019-1025-3#ref-CR38)]. A review conducted on the effect of environmental interventions reported positive results on dietary intake from environmental modification such as food labeling, health education and promotional materials (brochures and posters), expanding the availability of healthy products, and efficient food placement [[39](https://bmccardiovascdisord.biomedcentral.com/articles/10.1186/s12872-019-1025-3#ref-CR39)]. However, most interventions were multicomponent, so it is difficult to differentiate between the specific effect of environmental and individual level interventions.

We have some limitations. First, the nurses work in shifts of 8 h in the morning, afternoon or night. Their biological measures might be influenced by their shifts of the previous day. Second, we anticipate that their participation in the individual behavior component will be compromised. We will offer the behavior classes three times a day so that they can join according to their time availability. In addition, we will re-schedule the behavioral classes every other week based on their shift work. Third, we will collect the 24-h dietary recall on the days that are feasible to the participants. So, these may not be well representation of both week days and weekends. However, we will use the same strategy at all points of data collection. Therefore, the error will be non-differential to the cafeteria or behavioral intervention.

This pioneering study will investigate the effect of an environmental intervention to reduce cardio-metabolic risks and will estimate the added benefit of a proven individual-level dietary intervention for preventing cardio-metabolic risk. If the study demonstrates a significant effect, a scaled up approach could produce an important reduction in CVD burden through environmental and individual level prevention programs. The lessons learned may also be replicated in similar worksites in Nepal and translated to similar settings globally. The study needs to be tested in variety of workplace settings that are likely to represent different contexts that will be encountered at full scale. The study will provide the effectiveness of each level of intervention separately, which will be a vital information on what to focus in these settings in future. Advocacy, dialogue and planning with multiple stakeholders including the government and other partners would be necessary for scaling up the effective interventions at worksites to prevent cardio-metabolic risk. This study will provide the groundwork for it.

## Abbreviations

CHOD:

Cholesterol Oxidase

CONSORT:

Consolidated Standards of Reporting Trials

COPD:

Chronic Obstructive Pulmonary Disease

CVD:

Cardiovascular Disease

DH-KUH:

Dhulikhel Hospital - Kathmandu University Hospital

DPP:

Diabetes Prevention Program

GEE:

Generalized Estimating Equation

GPO:

Glycerol Phosphate Oxidase

HbA1c:

Glycated Hemoglobin

HDL:

High Density Lipoprotein

IDRS:

Indian Diabetes Risk Score

LDL:

Low Density Lipoprotein

LTF:

Lost To Follow up

MET:

Metabolic Equivalent of Task

NCD:

Non Communicable Disease

NIH:

National Institute of Health

RA:

Research Assistant

USA:

United States of America

## References

1. Basnyat B, Rajapaksa LC. Cardiovascular and infectious diseases in South Asia: the double whammy. BMJ. 2004;328(7443):781.
2. Dans A, Ng N, Varghese C, Tai ES, Firestone R, Bonita R. The rise of chronic non-communicable diseases in Southeast Asia: time for action. Lancet. 2011;377(9766):680–9.
3. Edelstein SL, Knowler WC, Bain RP, Andres R, Barrett-Connor EL, Dowse GK, Haffner SM, Pettitt DJ, Sorkin JD, Muller DC, Collins VR, Hamman RF. Predictors of progression from impaired glucose tolerance to NIDDM: an analysis of six prospective studies. Diabetes. 1997;46(4):701–10.
4. de Munter JS, Hu FB, Spiegelman D, Franz M, van Dam RM. Whole grain, bran, and germ intake and risk of type 2 diabetes: a prospective cohort study and systematic review. PLoS Med. 2007;4(8):e261.
5. LeFevre ML, Force USPST. Behavioral counseling to promote a healthful diet and physical activity for cardiovascular disease prevention in adults with cardiovascular risk factors: U.S. preventive services task Force recommendation statement. Ann Intern Med. 2014;161(8):587–93.
6. Lin JS, O'Connor E, Evans CV, Senger CA, Rowland MG, Groom HC. Behavioral counseling to promote a healthy lifestyle in persons with cardiovascular risk factors: a systematic review for the U.S. preventive services task Force. Ann Intern Med. 2014;161(8):568–78.
7. Anderson LM, Quinn TA, Glanz K, Ramirez G, Kahwati LC, Johnson DB, Buchanan LR, Archer WR, Chattopadhyay S, Kalra GP, Katz DL, TFoCP S. The effectiveness of worksite nutrition and physical activity interventions for controlling employee overweight and obesity: a systematic review. Am J Prev Med. 2009;37(4):340–57.
8. Conn VS, Hafdahl AR, Cooper PS, Brown LM, Lusk SL. Meta-analysis of workplace physical activity interventions. Am J Prev Med. 2009;37(4):330–9.
9. Carnethon M, Whitsel LP, Franklin BA, Kris-Etherton P, Milani R, Pratt CA, Wagner GR, Committee AHAAC. Prevention CoEa, disease CotKiC, council on nutrition PyAaM. Worksite wellness programs for cardiovascular disease prevention: a policy statement from the American Heart Association. Circulation. 2009;120(17):1725–41.
10. Kaspin LC, Gorman KM, Miller RM. Systematic review of employer-sponsored wellness strategies and their economic and health-related outcomes. Popul Health Manag. 2013;16(1):14–21.
11. Katz DL, O'Connell M, Yeh MC, Nawaz H, Njike V, Anderson LM, Cory S, Dietz W. Services TFoCP. Public health strategies for preventing and controlling overweight and obesity in school and worksite settings: a report on recommendations of the task Force on community preventive services. MMWR Recomm Rep. 2005;54(RR-10):1–12.
12. Matson-Koffman DM, Brownstein JN, Neiner JA, Greaney ML. A site-specific literature review of policy and environmental interventions that promote physical activity and nutrition for cardiovascular health: what works? Am J Health Promot. 2005;19(3):167–93.
13. CBS. Nepal Labor Force Survey. Thapathali, Kathmandu: Central Bureau of Statistics, 2008.
14. Mohan V, Deepa R, Deepa M, Somannavar S, Datta M. A simplified Indian diabetes risk score for screening for undiagnosed diabetic subjects. J Assoc Physicians India. 2005;53:759–63.
15. Hartung C, Lerer A, Anokwa Y, Tseng C, Brunette W, Borriello G. Open data kit: tools to build information services for developing regions. Proceedings of the 4th ACM/IEEE International Conference on Information and Communication Technologies and Development: ACM, 2010;18.
16. Bull FC, Maslin TS, Armstrong T. Global physical activity questionnaire (GPAQ): nine country reliability and validity study. J Phys Act Health. 2009;6(6):790–804.
17. Government of Nepal. Food Composition Table for Nepal 2012. Kathmandu, Nepal: Department of Food Technology and Quality Control, 2012.
18. SKUP. Afinion™ system for HbA1c. A system for measurement of B-haemoglobin A1c manufactured by Axis-Shield PoC AS, Norway. <https://skup.org/GetFile.ashx?fileid=470>. Accessed 24 Feb 2019.
19. DIALAB. Glucose Hexokinase 2 Reagents. Diagnostic reagent for quantitative in vitro determination of glucose in human serum, plasma, urine and capillary blood on photometric systems. DIALAB Produktion und Vertrieb von chemisch – technischen Produkten und Laborinstrumenten Gesellschaft m.b.H, Austria 2004.
20. DIALAB. Cholesterol HDL, Direct immunoinhibition 2 Reagent. Diagnostic reagent for quantitative in vitro determination of high density lipoprotein cholesterol (HDL-C) in human serum or plasma or photometric systems. DIALAB Produktion und Vertrieb von chemisch – technischen Produkten und Laborinstrumenten Gesellschaft m.b.H. Austria, 2013.
21. DIALAB. Triglycerides GPO-PAP with ATCS Single Reagent. Diagnostic Reagent for quantitative in vitro determination of Triglycerides in human serum or plasma on photometric systems. DIALAB Produktion und Vertrieb von chemisch – technischen Produkten und Laborinstrumenten Gesellschaft m.B.H.. Austria, 2015.
22. DIALAB. Cholesterol CHOD-PAP with ATCS Single Reagent. Diagnostic Reagent for Quantitative in Vitro Determination of Cholesterol in Serum or Plasma on Photometric Systems. DIALAB Produktion und Vertrieb von chemisch – technischen Produkten und Laborinstrumenten Gesellschaft m.b.H. Austria.
23. Fung TT, McCullough M, van Dam RM, Hu FB. A prospective study of overall diet quality and risk of type 2 diabetes in women. Diabetes Care. 2007;30(7):1753–7.
24. Group DPPDR. The diabetes prevention program (DPP): description of lifestyle intervention. Diabetes Care. 2002;25(12):2165–71.
25. Hussain SM, Oldenburg B, Wang Y, Zoungas S, Tonkin AM. Assessment of cardiovascular disease risk in south asian populations. Int J Vasc Med. 2013;2013:786801.
26. Turpin DL. CONSORT and QUOROM guidelines for reporting randomized clinical trials and systematic reviews. Am J Orthod Dentofac Orthop. 2005;128(6):681–5 discussion 686.
27. Zeger SL, Liang KY. Longitudinal data analysis for discrete and continuous outcomes. Biometrics. 1986;42(1):121–30.
28. Liu G, Liang KY. Sample size calculations for studies with correlated observations. Biometrics. 1997;53(3):937–47.
29. Weber MB, Ranjani H, Meyers GC, Mohan V, Narayan KM. A model of translational research for diabetes prevention in low and middle-income countries: the diabetes community lifestyle improvement program (D-CLIP) trial. Prim Care Diabetes. 2012;6(1):3–9.
30. Machin D, Campbell M, Tan S, Tan S. (n.d.). Sample size tables for clinical studies. 3rd ed. Oxford: Wiley; 2011.
31. Gutman SA, Murphy SL. Information commonly unreported in intervention effectiveness studies. Am J Occup Ther. 2012;66(1):7–10.
32. Eckel RH, Jakicic JM, Ard JD, de Jesus JM, Houston Miller N, Hubbard VS, Lee IM, Lichtenstein AH, Loria CM, Millen BE, Nonas CA, Sacks FM, Smith SC, Svetkey LP, Wadden TA, Yanovski SZ, Kendall KA, Morgan LC, Trisolini MG, Velasco G, Wnek J, Anderson JL, Halperin JL, Albert NM, Bozkurt B, Brindis RG, Curtis LH, DeMets D, Hochman JS, Kovacs RJ, Ohman EM, Pressler SJ, Sellke FW, Shen WK, Tomaselli GF, ACoCAHATFoP G. 2013 AHA/ACC guideline on lifestyle management to reduce cardiovascular risk: a report of the American College of Cardiology/American Heart Association task Force on practice guidelines. Circulation. 2014;129(25 Suppl 2):S76–99.
33. Riekert KA, Ockene JK. Pbert L. The handbook of health behavior change. New York: Springer Publishing Company; 2013.
34. Brawley LR, Rejeski WJ, King AC. Promoting physical activity for older adults: the challenges for changing behavior. Am J Prev Med. 2003;25(3 Suppl 2):172–83.
35. Groeneveld IF, Proper KI, van der Beek AJ, Hildebrandt VH, van Mechelen W. Lifestyle-focused interventions at the workplace to reduce the risk of cardiovascular disease--a systematic review. Scand J Work Environ Health. 2010;36(3):202–15.
36. Schröer S, Haupt J, Pieper C. Evidence-based lifestyle interventions in the workplace--an overview. Occup Med (Lond). 2014;64(1):8–12.
37. Heaney CA, Goetzel RZ. A review of health-related outcomes of multi-component worksite health promotion programs. Am J Health Promot. 1997;11(4):290–307.
38. Danna K, Griffin RW. Health and well-being in the workplace: a review and synthesis of the literature. J Manag. 1999;25(3):357–84.
39. Engbers LH, van Poppel MN, Paw MJCA, van Mechelen W. Worksite health promotion programs with environmental changes: a systematic review. Am J Prev Med. 2005;29(1):61–70.
