# Supplementary figures and images for: Effects of a dietary intervention on cardiometabolic risk and food consumption in a workplace
Source: PLoS One. 2024 Apr 24;19(4):e0301826. doi: 10.1371/journal.pone.0301826 (PMC11042715; doi:10.1371/journal.pone.0301826)

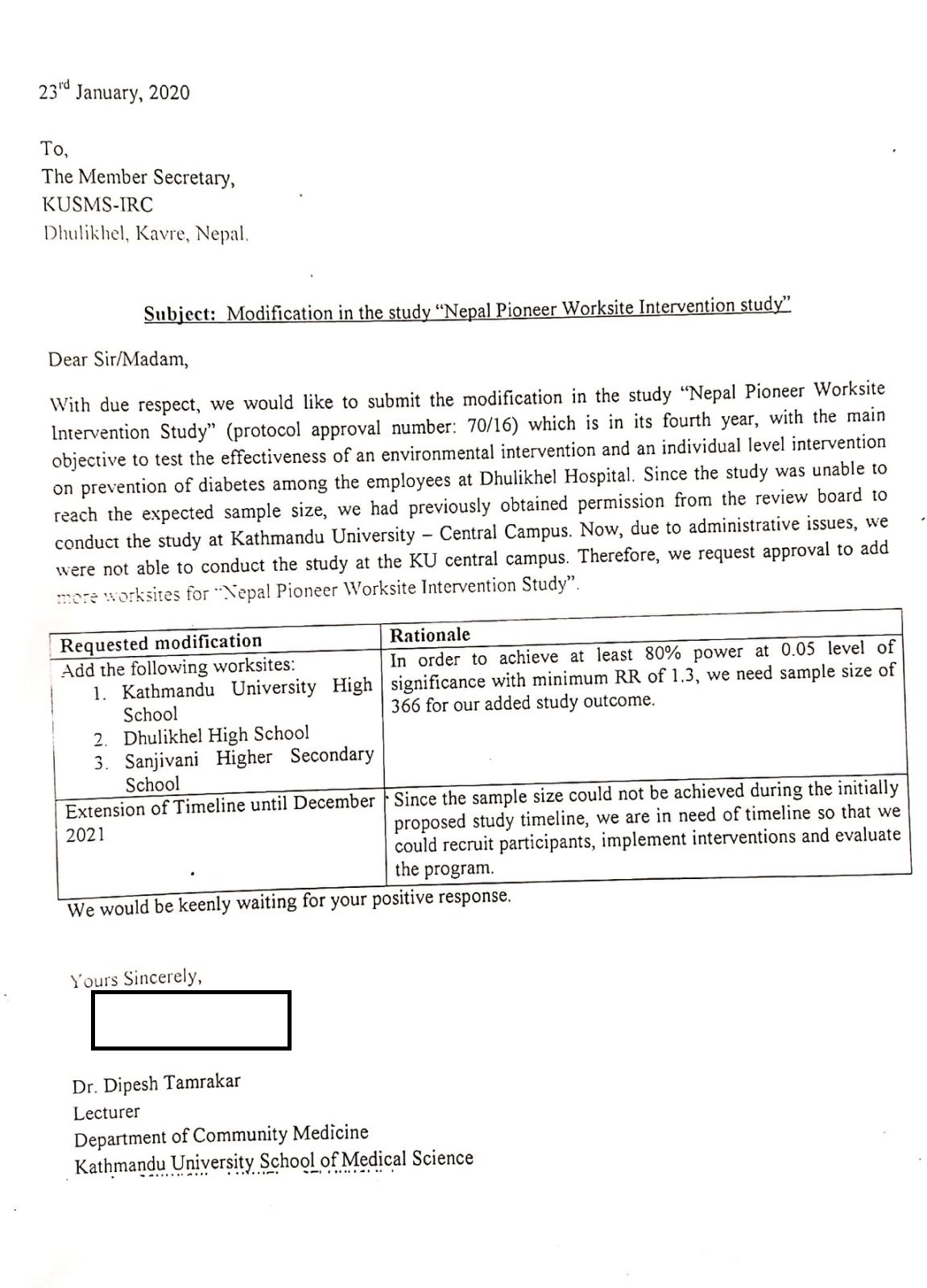

Supplement: S4 File — (JPG) [file pone.0301826.s007.jpg]
