# Supplementary material for: Effects of a dietary intervention on cardiometabolic risk and food consumption in a workplace
Source: PLoS One. 2024 Apr 24;19(4):e0301826. doi: 10.1371/journal.pone.0301826 (PMC11042715; doi:10.1371/journal.pone.0301826)
Supplement: S6 File — (DOCX) [file pone.0301826.s009.docx]

Harvard T.H. Chan School of Public Health

Office of Human Research Administration

90 Smith Street, 3rd Floor Boston, MA 02120

Federalwide Assurance FWA00002642

**Notification of Modification Approval**

| July 9, 2018  Walter Willett stdls@hsph.harvard.edu |  |
| --- | --- |
| **Protocol Title:** | Nepal Pioneer Worksite Intervention Study |
| **Principal Investigator:** | Walter Willett |
| **Protocol #:** | IRB16-0301 |
| **Submission #:** | MOD16-0301-05 |
| **Funding Source:** | NIH/NIEHS- 5DP1ES025459-04 (Active) |
| **Review Date:** | 7/9/2018 |
| **MOD Effective Date:** | 7/9/2018 |
| **Expiration Date:** | 4/24/2019 |
| **IRB Review Type:** | Expedited |
| **IRB Review Action:** | Approved |

The Institutional Review Board (IRB) of the Harvard T.H. Chan School of Public Health approved this Modification. **Please note that the approval for this protocol will lapse on 4/24/2019.**

Please upload to the Local Site Documents page an email from Dr. Walter Willett confirming he agrees to take over this project.

This approval includes the following:

- Modifications as described in MOD16-0301-05 o Changes in Principal Investigator – From Donna Spiegelman to Walter Willett.

o Revised Research Protocol – To reflect the change in PI.

- IRB Protocol: nepal pioneer PROTOCOL DH_June29_clean.doc (4)

Please contact me at 617.432.5132 or kninsala@hsph.harvard.edu with any questions.

Sincerely,

Keren-Nicole Insalaco

Sr. IRB Review Specialist

University Area IRB [http://cuhs.harvard.edu](http://cuhs.harvard.edu/)

Longwood Medical Area IRB <http://www.hsph.harvard.edu/ohra/>

Template v04/06/2018
