# Supplementary material for: Effects of a dietary intervention on cardiometabolic risk and food consumption in a workplace
Source: PLoS One. 2024 Apr 24;19(4):e0301826. doi: 10.1371/journal.pone.0301826 (PMC11042715; doi:10.1371/journal.pone.0301826)
Supplement: S1 File — (DOCX) [file pone.0301826.s004.docx]

KATHMANDU UNIVERSITY

# SCHOOL OF MEDICAL SCIENCES

Date: August 19, 2016

To,

Dr. Archana Shrestha

Department of Epidemiology

Harvard TH Chan School of Public Health Boston, MA

To,

Dr. Dipesh Tamrakar

Department of Community Medicine

Kathmandu University School of Medical Sciences Dhulikhel, Kavre

Dear Dr. Archana & Dr Dipesh

The Institutional Review Committee of Kathmandu University School of Medical Sciences/Dhulikhel Hospital (IRC-KUSMS) reviewed and discussed your application to conduct study entitled "Nepal Pioneer Worksite Intervention Study" on 10^th^ July 2016.

The Committee has approved the protocol.

Your protocol approval number is: 70/16

The IRC-KUSMS expects to be informed about the progress of the study, any changes in the protocol and patient information/informed consent. A copy of the final report should be submitted to IRC-KUSMS.

With best regards,

Dr. Dipak Shrestha

Member Secretary, IRC-KUSMS
