# Supplementary material for: Effects of a dietary intervention on cardiometabolic risk and food consumption in a workplace
Source: PLoS One. 2024 Apr 24;19(4):e0301826. doi: 10.1371/journal.pone.0301826 (PMC11042715; doi:10.1371/journal.pone.0301826)
Supplement: S5 File — (DOCX) [file pone.0301826.s008.docx]

KATHMANDU UNIVERSITY

# SCHOOL OF MEDICAL SCIENCES

Date: May 21, 2018

To,

Dr. Archana Shrestha

Department of Epidemiology

Harvard T.H. Chain School of Public Health Boston, MA, USA

Dear Dr. Shrestha

The Institutional Review Committee of Kathmandu University School of Medical Sciences/Dhulikhel Hospital (IRC-KUSMS) has received your application for modification and approved to conduct study entitled "Nepal Pioneer worksite intervention study" on 18^th^ May, 2018. This approval shall remain in effect until 20^th^ May 2019.

The following personnel are approved to perform research activities on this protocol:

| l. Dr. Archana Shrestha | Principal Investigator |
| --- | --- |
| 2. Dr. Dipesh Tamrakar | Co-Principal Investigator |
| 3. Ms. Donna Spiegelman | Co- Investigator |
| 4. Dr. Biraj Man Karmacharya | Co-Investigator |
| 5. Mr. Rajendra Dev Bhatt | Co-Investigator |

Your protocol approval number is: 70/16

The IRC-KUSMS expects to be informed about the progress of the study, any changes in the protocol and patient information/informed consent. A copy of the final report should be submitted to IRC-KUSMS.

With best regards,

Dr. Rajeev Shrestha

Member Secretary, IRC-KUSMS
